# Are standing osmotic gradients the main driver of cerebrospinal fluid production? A computational analysis

**DOI:** 10.1186/s12987-023-00419-2

**Published:** 2023-03-13

**Authors:** Pooya Razzaghi Khamesi, Vasileios Charitatos, Eva K. Heerfordt, Nanna MacAulay, Vartan Kurtcuoglu

**Affiliations:** 1 The Interface Group, Institute of Physiology, University of Zurich, Winterthurerstrasse 190, 8057 Zurich, Switzerland; 2 Department of Neuroscience, University of Copenhagen, Copenhagen, Denmark; 3 Zurich Center for Integrative Human Physiology, University of Zurich, Zurich, Switzerland; 4 Neuroscience Center Zurich, University of Zurich, Zurich, Switzerland

**Keywords:** CSF production, Choroid plexus, Computational modeling

## Abstract

**Background:**

The mechanisms of cerebrospinal fluid (CSF) production by the ventricular choroid plexus (ChP) have not been fully deciphered. One prominent hypothesized mechanism is trans-epithelial water transport mediated by accumulation of solutes at the luminal ChP membrane that produces local osmotic gradients. However, this standing osmotic gradient hypothesis has not been systematically tested.

**Methods:**

To assess the plausibility of the standing gradient mechanism serving as the main driver of CSF production by the ChP, we developed a three-dimensional (3D) and a one-dimensional (1D) computational model to quantitatively describe the associated processes in the rat ChP inter-microvillar spaces and in CSF pools between macroscopic ChP folds (1D only). The computationally expensive 3D model was used to examine the applicability of the 1D model for hypothesis testing. The 1D model was employed to predict the rate of CSF produced by the standing gradient mechanism for 200,000 parameter permutations. Model parameter values for each permutation were chosen by random sampling from distributions derived from published experimental data.

**Results:**

Both models predict that the CSF production rate by the standing osmotic gradient mechanism is below 10% of experimentally measured values that reflect the contribution of all actual production mechanisms. The 1D model indicates that increasing the size of CSF pools between ChP folds, where diffusion dominates solute transport, would increase the contribution of the standing gradient mechanism to CSF production.

**Conclusions:**

The models suggest that the effect of standing osmotic gradients is too small to contribute substantially to CSF production. ChP motion and movement of CSF in the ventricles, which are not accounted for in the models, would further reduce this effect, making it unlikely that standing osmotic gradients are the main drivers of CSF production.

**Supplementary Information:**

The online version contains supplementary material available at 10.1186/s12987-023-00419-2.

## Introduction

Cerebrospinal fluid (CSF) is produced primarily by the choroid plexus (ChP) of the four cerebral ventricles [1]. There is no consensus on how CSF is generated, but several mechanisms have been proposed, including hydrostatic pressure difference between blood and CSF [2], water transport through blood-CSF barrier tight junctions (by mechano-diffusion [3], electro-diffusion [4], or claudin mediation [5]), or by molecular ion transporter-mediated water translocation, e.g., through the Na^+^-K^+^-2Cl^−^ cotransporter 1 (NKCC1), Na^+^-HCO_3_^−^ cotransporter (NBCe2), and Na^+^-K^+^-ATPase [6]. However, a prevalent hypothesis is that the ChP secretes CSF by osmosis, with water following an osmotic gradient produced by the transport of ions across the ChP epithelium [7]. This mechanism requires sufficient water permeability, along with either a global osmotic gradient across the ChP or local gradients over the epithelium. Global gradients are either small or nonexistent, with CSF reported to be either slightly hyperosmolar with respect to blood (approximately 5 mOsm) [8–11] or isosmolar [6, 12].

The origin of the idea of local osmotic gradients as the driving force behind CSF production can be traced back to experiments on water transport across the intestinal epithelium [13]. Water and solute transport from the luminal (gut) side to the serosal (blood) side was found to be a function of, primarily, cellular activity rather than a bulk osmotic pressure difference between the two sides [13]. To explain this, Diamond and Bossert proposed what came to be known as the *standing gradient (SG) model* [14], which was based on the work of Curran and MacIntosh who had proposed a three-compartment model of epithelial water transport [15].

Diamond and Bossert considered the intracellular and the lateral intercellular spaces as the first and the second compartment, respectively [16]. They assumed these to be separated by a selectively permeable membrane, through which solutes are actively transported from the first to the second compartment, where they produce a locally elevated solute concentration level. The ensuing local osmotic gradient across the membrane then drives water from the first to the second compartment, increasing the hydrostatic pressure therein. Consequently, the solution is transferred through an imaginary, fully permeable membrane to the third compartment, which is the basal tissue between the epithelial cells and the capillary blood vessel. The tissue was considered to have the same bulk osmolarity as the epithelial intracellular space.

Unlike the intestinal epithelium, which is “absorptive” in nature, the ChP epithelium is “secretory,” meaning that for ChP, net water flux is from the basolateral to the luminal side, i.e., from blood to the CSF space. While in absorptive epithelia a local increase in osmolyte concentration in the lateral intercellular space may serve as a driving force for water transport, in secretory epithelia, such a concentration gradient would oppose it. With the epithelial tight junctions localized on the luminal side in the ChP, the lateral intercellular spaces are on the ‘wrong’ side to enable local osmotic gradient-driven fluid transfer [1]. However, having observed the movement of water from ChP against a bulk concentration gradient, Pollay suggested that a favorable gradient could still be present, produced by increased osmolyte concentration at the luminal membrane of the ChP between microvilli, which are finger-like protrusions of the plasma membrane [17]. Thus, local osmotic gradients in the ChP inter-microvillar spaces could be drivers of CSF production.

Since direct experimental assessment of the SG hypothesis is difficult, mathematical models have been used to aid in its evaluation. Most of this work has been done for absorptive epithelia: for example, Sackin and Boulpaep [18] suggested that slight osmolarity differences (below the limit of detection) between interstitial fluid and blood in *Necturus* renal proximal tubule could be responsible for the observed quasi-isotonic water reabsorption. Consequently, there would be no need for an osmotic gradient in the lateral intercellular space. Schafer et al. [19] came to the same conclusion with their model for the rabbit proximal tubule. Hill argued that an abnormally high permeability would be required for SG-based isosmotic water transport in leaky epithelia [20, 21]. However, Diamond argued that these studies do not provide direct indications against the SG mechanism and called for further experimental evidence [22]. Pedley and Fischbarg [23] used a model to interpret the experimental results of Wright et al. [24] on water flux across the rabbit gallbladder epithelium, concluding that the experimental data were inconsistent with the SG model. The structural difference between the secretory ChP epithelium and absorptive epithelia requires a separate assessment of the plausibility of the SG hypothesis for the brain. We recently showed using a 1D computational model that—with the employed set of parameter values—the SG mechanism in the intermicrovillar space could not account for the CSF produced by the ChP [6]. However, the employed model did not take into account the possible effect of stagnant pools of CSF within ChP infoldings.

One aspect common to the mentioned models is their high sensitivity to input parameters and boundary conditions such as solute–solvent transport coupling at cellular membranes. This has blunted the impact of the corresponding studies on the scientific discourse. Therefore, models covering large anatomic and physiologic parameter ranges are required to allow for a more robust assessment of the SG hypothesis. In the current study, we tested whether standing osmotic gradients at the surface of the ChP epithelium could be the predominant factor for CSF production by the ChP in rats. To this end, we developed a one-dimensional (1D) computational model to describe the conjugate fluid and solute transport within a subunit of inter-microvillar space (defined as a functional unit, FU) and between macroscopic ChP folds, under the assumption that standing osmotic gradients are the only mechanism contributing to CSF production. We then compared the calculated CSF production rate to measured rates of CSF production, which include the contributions of all actual mechanisms. We emphasize that, unlike the original SG model, we did not limit solute transport to the bottom 10% of the FU [14], but considered solute influx along the entire length of the inter-microvillar space. Given the wide range of values reported for the relevant anatomic and biophysical parameters and the unknown sensitivity of the CSF production rate to variations in these, we performed computations with approximately 200,000 parameter value permutations. To avoid bias against the SG hypothesis, we selected a general model setup that would over- rather than underestimate the contribution of standing gradients to CSF production. Furthermore, to confirm the applicability of the 1D model for hypothesis testing, we also developed and deployed a separate three-dimensional (3D) model of inter-microvillar fluid and solute transport.

## Methods

### Model domain

**Fig. 1:**
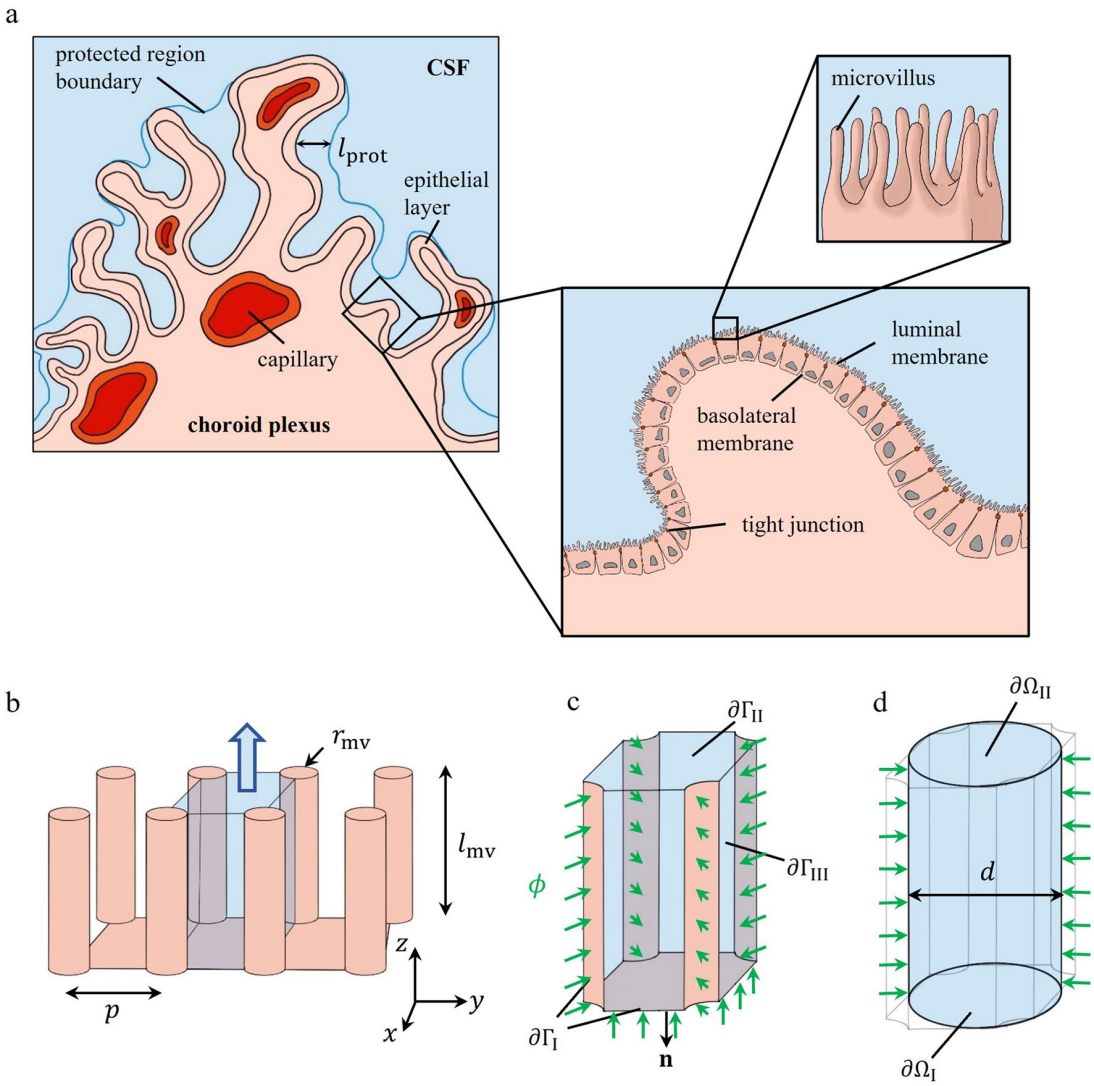
Schematic representation of the ChP and derivation of the computational domain. **a** Macrostructure of the ChP containing capillaries in the stroma and epithelial cells on the outer surface of it. Protected pools of CSF are within a distance of ${l}_{\text{prot}}$ from the epithelial surface, as shown in the main panel. The first inset shows the two main epithelial cell borders (basolateral and luminal membranes), separated by tight junctions. The second inset shows epithelial microvilli, in between which standing osmotic gradients can be present. **b** Simplified representation of the microvillar zone with homogeneously distributed cylindrical microvilli (radius ${r}_{\text{mv}}$ and length ${l}_{\text{mv}}$), spaced $p$ apart from each other. A FU (blue shaded region) with the net water flow direction along the z-axis is shown. **c** Isolated FU with the 3D computational domain in blue and defined boundaries. Solutes are injected with a flux of $\phi$ into the FU, normal to the $\partial {\Gamma }_{\text{I}}$ boundary, which corresponds to the luminal membrane. $\partial {\Gamma }_{\text{II}}$ and $\partial {\Gamma }_{\text{III}}$ show the opening to the protected pool and adjacent FUs, respectively. **d** Simplified 1D geometry of the FU (a cylinder with hydraulic diameter of $d$) with its boundaries. $\partial {\Omega }_{\text{I}}$ and $\partial {\Omega }_{\text{II}}$ are the FU base and tip, respectively. In contrast to the 3D model, solute injection is applied through the governing equations

We simplified the ChP luminal membrane to a surface covered with homogeneously distributed cylindrical pins representing microvilli as shown in Fig. 1. To account for the convoluted surface of the ChP, which produces pools shielded from the CSF movement at the center of the ventricles, we defined a protected area extending from the tip of the microvilli to a distance of ${l}_{\text{prot}}$ away from them (illustrated in Figs. 1, 3). Within the protected area, only flow caused by local CSF production contributes to solute convection.

Since the microvilli are arranged in a recurring pattern, we defined a representative functional unit (FU) as the space between four adjacent microvilli (Fig. 1b, c). Microvillar radius and length are ${r}_{\text{mv}}$ and ${l}_{\text{mv}}$, respectively. The microvillar separation distance, $p$, and the number of FUs, $N$, are given by

1 $$p=\sqrt{\frac{1}{\sigma }}$$

and

2 $$N=\sigma \cdot {A}_{\text{app}}$$

respectively, where $\sigma$ is the surface density of microvilli and ${A}_{\text{app}}$ is the apparent luminal surface area, i.e., the surface area of the ChP without extension by microvilli.

To derive the 1D model, we simplified the FU to a cylindrical channel with equivalent hydraulic diameter, $d$, as shown in Fig. 1d, where

3 $$d=\frac{4{A}_{\text{CS}}}{{P}_{\text{CS}}}=\frac{{p}^{2}-\pi {r}_{\text{mv}}^{2}}{p+\left(\frac{\pi }{2}-2\right){r}_{\text{mv}}}$$

Here, ${A}_{\text{CS}}$ and ${P}_{\text{CS}}$ are the cross-sectional area and perimeter, respectively, of the original FU.

### One-dimensional standing gradient model

The transport of fluid and solutes in the FU are coupled. Solute transport is governed by diffusion and convection, whereas fluid transport is driven by trans-membrane solute concentration differences. The fluid velocity $u\left(z\right)$ and solute concentration $C\left(z\right)$ along the longitudinal axis (z-axis) of the FU, as shown in Fig. 1d, are determined by Eqs. (4)–(8). This set of equations describes the conjugate transport of solute and water through the intermicrovillar space in the longitudinal direction.

4 $$\frac{4\phi \left(z\right)}{\rho d}+D\frac{{\text{d}}^{2}C}{\text{d}{z}^{2}}-C\left(z\right)\frac{\text{d}u}{\text{dz}}-u\left(z\right)\frac{\text{d}C}{\text{dz}}=0$$

5 $$\frac{\text{d}u}{\text{dz}}=\frac{4{L}_{\text{p}}}{d}\left[C\left(z\right)-{C}_{0}\right]$$

6 $$\frac{\text{d}C}{\text{dz}}=0 \quad \text{On }\,\partial {\Omega }_{\text{I}} (z=0)$$

7 $$u=0 \quad\text{On }\,\partial {\Omega }_{\text{I}} (z=0)$$

8 $$D\left(1-{e}^{\frac{u}{D}{l}_{\text{prot}}}\right)\frac{\text{d}C}{\text{dz}}-u\left(C-{C}_{0}\right)=0 \quad \text{On }\,\partial {\Omega }_{\text{II}} (z={l}_{\text{mv}})$$

Here, $\phi \left(z\right)$ is the local solute flux, $\rho$ is CSF density, and $D$ is the solute diffusion coefficient in CSF. ${L}_{\text{p}}$ is the water permeability of the luminal membrane and ${C}_{0}$ is the bulk ventricular CSF osmolality. Equations (4) and (5) describe the solute mass balance in the FU and the osmotic water transport through the luminal FU surface, respectively. They correspond to the equations of the original standing gradient model [14]. The boundary conditions represented by Eqs. (6) and (7) indicate, respectively, that the base of the FU is impermeable to solutes and fluid. This is necessary because of the single-dimensional characteristic of the model. However, since inflow through the FU base may occur in reality, solute flux through the lateral boundary is adapted to account for it (see Eq. (9)). Equation (8) represents the boundary condition at the FU tip, where the relative contributions of convective and diffusive transport of solutes are balanced as a function of the protected length (see Additional file 1). In the case of zero protected length, the concentration at the tip of the FU corresponds to bulk solute concentration, a condition considered in a previous model [6].

The solute flux used in Eq. (4) is calculated from the measured CSF production rate ${Q}_{\text{meas}}$ according to Eq. (9). While production may vary in time, experimentally determined ${Q}_{\text{meas}}$ and ${C}_{0}$ are time-averaged quantities. Therefore, steady-state forms of the governing equations and boundary conditions were employed (Eqs. (4)–(8)).

9 $$\phi \left(z\right)=\frac{{\rho Q}_{\text{meas}}{C}_{0}}{N(\pi d{l}_{\text{mv}}+\frac{\pi {d}^{2}}{4})}\cdot (1+{f}_{\text{b}})$$

Here, ${f}_{\text{b}}$ is a factor applied to the first $d/2{l}_{\text{mv}}$ of the FU length to correct for the lack of solute flux from the FU base. We chose the length of the flux-corrected section based on the FU aspect ratio to preempt potential numerical instabilities arising from a big jump in $\phi (z)$. We note that the choice of a specific length has little effect on the calculated CSF production rate. ${f}_{\text{b}}$ has a value of 0.5 within the modified section (corresponding to the area ratio of the base to the side of this section) and zero everywhere else.

### Three-dimensional standing gradient model

The 3D model considers coupled fluid and solute transport, taking into account viscous forces that were not included in the 1D model. The fluid velocity and pressure and the solute concentration distributions are governed by Eqs. (10)–(15). This set of equations determines the same quantities as that underlying the 1D model, but does so taking into account CSF flow in all directions (rather than only in the longitudinal direction) in a more realistic FU geometry.

10 $$\nabla \cdot \mathbf{V}=0$$

11 $$\rho \left(\mathbf{V}\cdot \nabla \right)\mathbf{V}= -\nabla P+\mu {\nabla }^{2}\mathbf{V}$$

12 $$\rho \mathbf{V}\cdot \nabla C=\rho D{\nabla }^{2}C$$

Here, $\mathbf{V}=\left({u}_{x},{u}_{y},{u}_{z}\right)$ is the fluid velocity vector, $P$ is fluid pressure, and $\mu$ is the dynamic viscosity of CSF.

13 $$\mathbf{V}={L}_{\text{p}}\left(C-{C}_{0}\right)\mathbf{n}$$

14 $$\rho \left(\mathbf{V}C-D\nabla C\right)\cdot \mathbf{n}=\phi$$

15 $$\phi =\frac{{\rho Q}_{\text{meas}}{C}_{0}}{N(2\pi {r}_{\text{mv}}{l}_{\text{mv}}+{p}^{2})}$$

Equations (10) and (11) describe, respectively, mass conservation of the fluid in the FU and the balance between pressure and viscous forces on the fluid. Solute transport via advection and diffusion while conserving solute mass is described by Eq. (12). The transport of fluid and solute is assumed to take place under steady-state conditions. Fluid and solute enter the FU perpendicularly from the luminal surface (Fig. 1c), with the corresponding velocity and flux given by Eqs. (13) and (14), respectively. The solute flux in the 3D model does not require the correction factor ${f}_{\text{b}}$, since flux through the FU base is taken into the account through the boundary condition in Eq. (14). We note that while the overall amount of solute entering the FU is the same in the 1D and 3D models, the flux is different (Eq. (15)). This is because the surfaces through which the solutes enter are not identical. At the tip of the FU, CSF has zero gauge-pressure and the same solute concentration as bulk ventricular CSF. Symmetry conditions are applied on boundaries to adjacent FUs, as the repetitive pattern of FU distribution implies zero net exchange of fluid and solute between them. The CSF production rate by the standing gradient mechanism is computed by integrating the velocity field on the entire ChP luminal surface.

### Choice of model parameters

For verification of our 1D model against the original implementation of the standing gradient one [14], we employed the same model parameters as used in the original study by Diamond and Bossert (Table 1). We emphasize that this model parameter set was employed for comparison purposes only, since Diamond and Bossert did not provide results for secretory epithelia. Our subsequent calculations relied on parameters reflective of the ChP.

**Table 1:** Parameter values used for comparing results of the current 1D SG model to the original standing gradient one

Parameters	Symbol	Value
Microvillus length (µm)	${l}_{\text{mv}}$	100
Hydraulic diameter (µm)	$d$	0.1
Protected length (µm)	${l}_{\text{prot}}$	0
Diffusion coefficient (cm^2^/s)	$D$	10^–5^
Luminal membrane permeability (cm/s Osm)	${L}_{\text{p}}$	2 · 10^–4^
Bulk solute concentration (Osm)	${C}_{0}$	0.3
CSF density (g/mL)	$\rho$	1.00
Solute flux (mmol/s · cm^2^)	$\phi$	10^–5^ for $z\le 0.1 {l}_{\text{mv}}$ 0 for $z>0.1 {l}_{\text{mv}}$

The solute flux depends on the location in the FU. These values correspond to those originally used by Diamond and Bossert and are not representative of the ChP

**Fig. 2:**
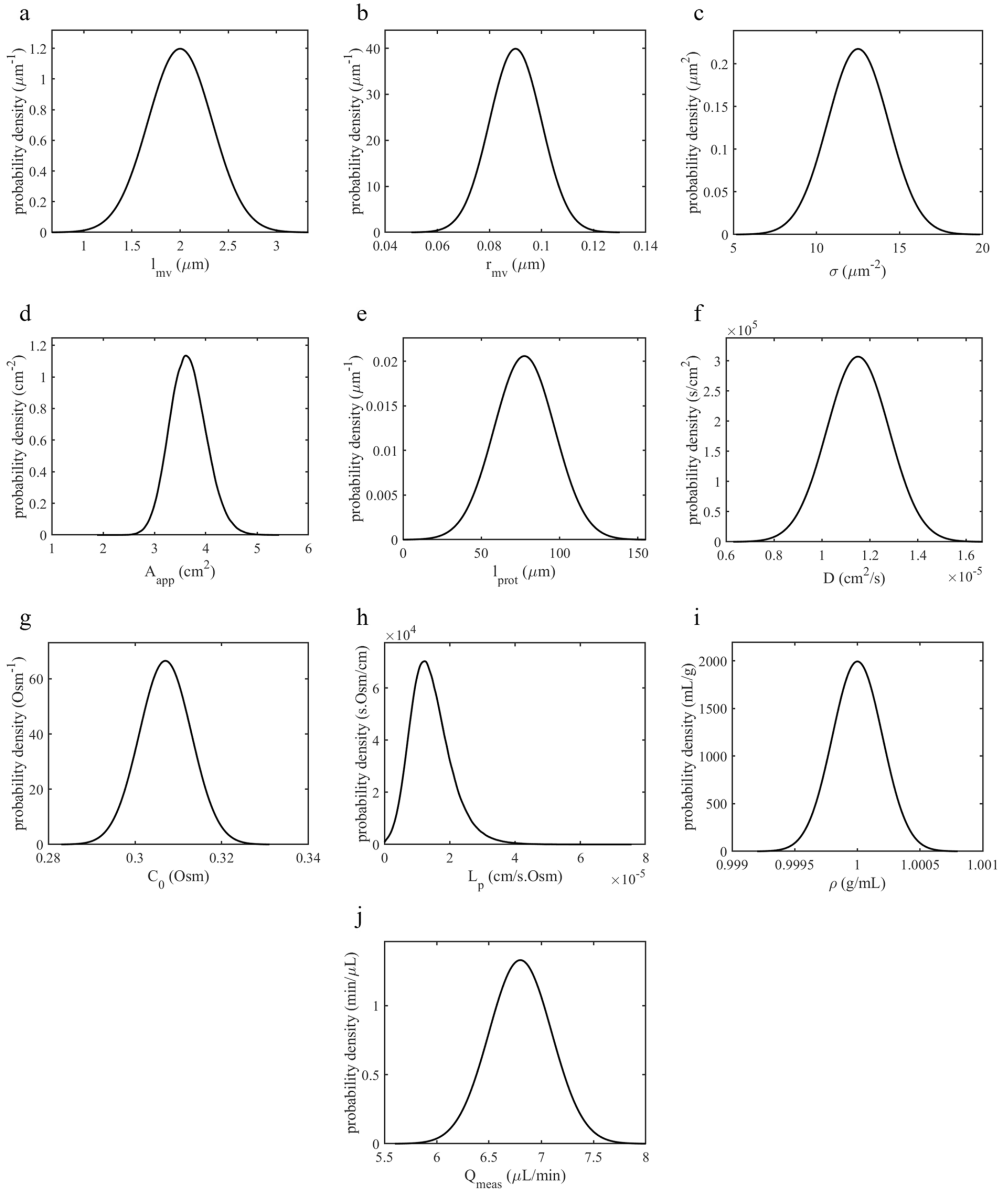
Probability density functions of the model parameters described in Table 2

**Fig. 3:**
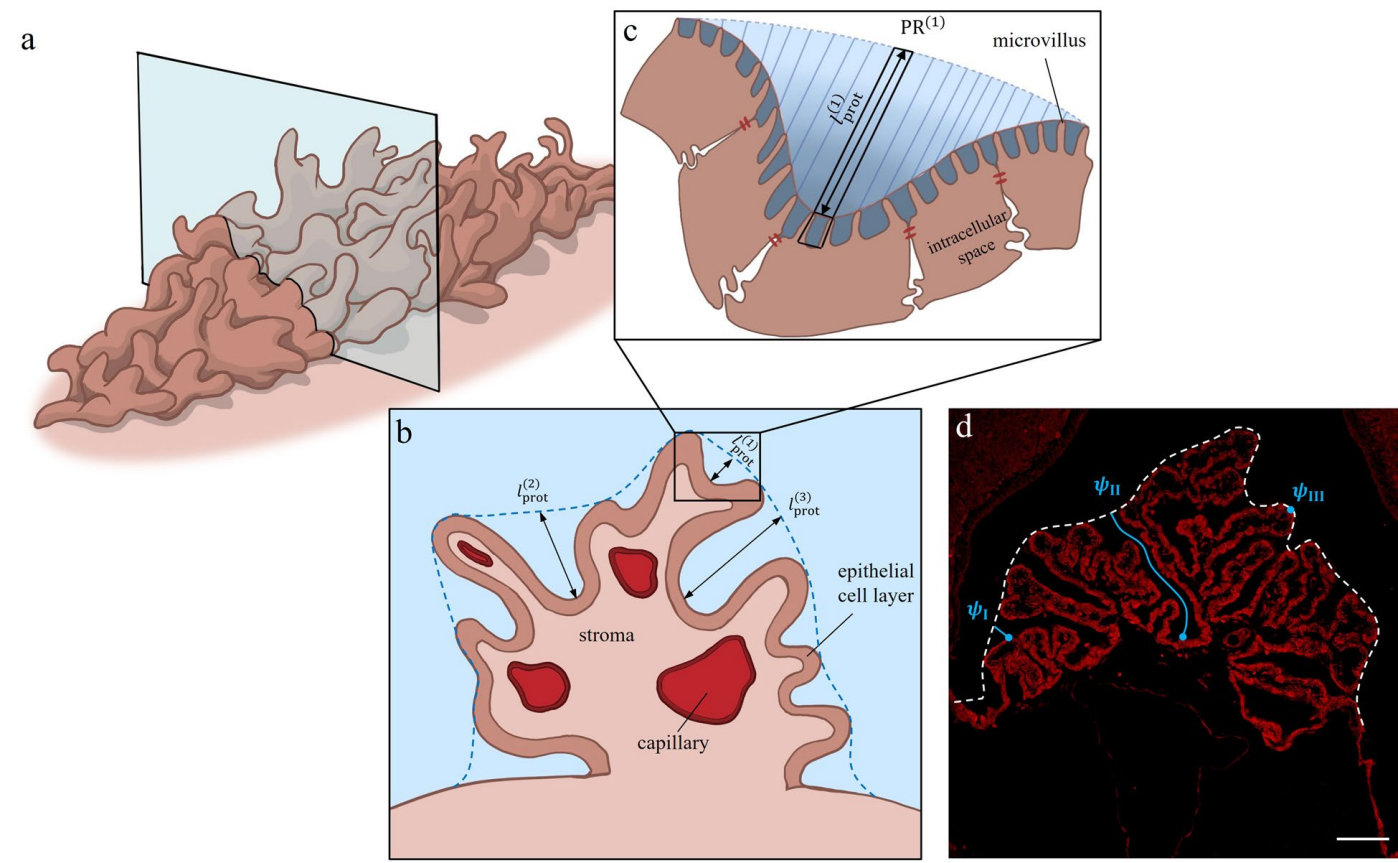
The choroid plexus with protected regions between its folds. **a** 3D schematic representation of the ChP with a section plane. **b** Cross-section of the ChP (from panel **a**). Capillary blood vessels, stroma, and the layer of epithelial cells forming the interface to ventricular CSF are shown. The blue dashed line delineates the boundary between protected regions and bulk CSF. The protected lengths (${l}_{\text{prot}}$) at three exemplary locations are indicated with black arrows. **c** Magnified view of a protected region and the neighboring ChP epithelium. The intermicrovillar spaces (dark blue) and their segments of the protected region (PR, in blue) are shown. **d** Laser scanning confocal microscopy image of a rat lateral ventricular ChP section. The white dashed line delineates the border between protected regions and bulk CSF. The blue points mark three exemplary positions on the epithelium at different distances from the outer surface of the ChP. The blue lines indicate the paths ($\psi$) along which the protected length for each position is determined. Scale bar: 75 µm

To test whether standing osmotic gradients in the ChP inter-microvillar spaces can account for most of the ventricular CSF production, we considered distributions of parameter values as illustrated in Fig. 2 and described in Table 2. This was done to account for uncertainties in the experimental determination of the respective parameters, as well as to reflect differences in experimental settings in cases where multiple sources of data were available. The distributions were established by assuming normal distributions and then calculating the mean and standard deviation of data reported in the literature. The distribution chosen for the diffusion coefficient of solutes in CSF is representative of Na^+^, the most relevant ion for the SG mechanism. We computed the distribution of the ChP apparent area and luminal membrane permeability based on the distribution of other related parameters (see Additional file 1). A precise evaluation of the protected length distribution over the ChP luminal surface would necessitate a 3D in vivo scan of the entire rat ChP at single-cell resolution, followed by a cell-by-cell calculation of the distance from the epithelium to the bulk CSF region (see Fig. 3d for an illustration of different protected lengths). Given that such data are currently unavailable, we relied on the Waxholm Space Rat Brain Atlas (v4, RRID: SCR_017124) [25], on measurements of ChP mass and volume [26–28], and on a geometric analysis to estimate ${l}_{\text{prot}}$ as detailed in Additional file 1. This yielded an upper limit of 77.5 µm for the average protected length. To be conservative—longer ${l}_{\text{prot}}$ favors the SG mechanism—we chose this upper limit as the center value for a normal distribution that extends to 155 µm. For comparison of the 1D to the 3D model, we used three sets of parameter values (Table 3). One of these corresponds to the parameter set that yields the mean value of CSF production rate as per the 1D model with zero protected length. The other two sets correspond to those yielding, respectively, mean ± standard deviation production rate.

**Table 2:** Parameters used in the 1D model with the corresponding references

Parameters	Symbol	Mean	SD	Distribution	References
Microvillus length (µm)	${l}_{\text{mv}}$	2	0.33		[1]
Microvillus radius (µm)	${r}_{\text{mv}}$	0.09	0.01		[6]
Microvilli surface density (1/µm^2^)	$\sigma$	12.5	1.83		[1]
ChP apparent area (cm^2^)	${A}_{\text{app}}$	3.66	0.35	Non-normal	[26–28]
Protected length (µm)	${l}_{\text{prot}}$	77.5	19.4		[25, 26, 28]
Diffusion coefficient (cm^2^/s)	$D$	1.15 × 10^–5^	1.3 × 10^–6^		[34, 35]
Bulk solute concentration (Osm)	${C}_{0}$	0.307	0.006		[36]
Luminal membrane permeability (cm/s · Osm)	${L}_{\text{p}}$	1.44 × 10^–5^	6.42 × 10^–6^	Non-normal	[6]
CSF density (g/mL)	$\rho$	1.00	0.0002		[37]
Measured CSF production rate (µL/min)	${Q}_{\text{meas}}$	6.8	0.3		[6]

For the 1D parameter values, references are indicated, and mean and standard deviation (SD) given. All directly measured parameters are assumed to be normally distributed. Some parameters calculated as a function of directly measured ones have non-normal distributions (indicated in Additional file 1: Table S1)

**Table 3:** 3D model parameters, obtained from the 1D model with zero protected length

Parameters	Symbol	Value		
		Mean − SD	Mean	Mean + SD
Microvillus length (µm)	${l}_{\text{mv}}$	2.329	1.875	1.959
Microvillus radius (µm)	${r}_{\text{mv}}$	0.087	0.104	0.098
Microvilli surface density (1/µm^2^)	$\sigma$	10.0	15.3	12.6
ChP apparent area (cm^2^)	${A}_{\text{app}}$	3.54	3.85	3.87
Protected length (µm)	${l}_{\text{prot}}$	0	0	0
Diffusion coefficient (cm^2^/s)	$D$	1.12 · 10^–5^	1.06 · 10^–5^	1.08 · 10^–6^
Bulk solute concentration (Osm)	${C}_{0}$	0.2967	0.3129	0.3041
Luminal membrane permeability (cm/s · Osm)	${L}_{\text{p}}$	5.03 · 10^–6^	9.82 · 10^–6^	2.17 · 10^–5^
CSF density (g/mL)	$\rho$	1.0002	1.0001	1.0002
Measured CSF production rate (µL/min)	${Q}_{\text{meas}}$	7.071	7.346	7.072
CSF viscosity (Pa · s)	$\mu$	8.9 · 10^–4^	8.9 · 10^–4^	8.9 · 10^–4^

The three columns (mean − SD, mean, and mean + SD) represent the set of parameter values that yield the mean CSF production rate (center column) or its mean plus or minus standard deviation value (right and left columns, respectively). Note that CSF viscosity is not considered in the 1D model

### Numerical procedure

Equations (4) and (5) were solved in MATLAB R2020b using bvp4c, a fourth-order numerically accurate finite difference solver for systems of ordinary differential equations. Equations (10)–(12) were solved using the finite-volume computational fluid dynamics software ANSYS Fluent on unstructured grids consisting of approximately 131,000 to 172,000 cells (Table 3). A second-order scheme was used for momentum and transport equation discretization. A grid independence study was performed to ensure that the calculated CSF production rate was not unduly influenced by the choice of the computational mesh.

To calculate the CSF production rate with the 1D model based on the parameter distributions described in Table 2, approximately 200,000 parameter value sets were randomly chosen while considering the likelihood of each choice. To this end, we used the Latin hypercube sampling technique [29], which generates random points within equal probability intervals of the considered distribution function, and thereby ensures proper coverage of the stochastic space. An independence study for the number of sampling points was performed to ensure that the calculated CSF production rate distribution did not change with more samples.

### Choroid plexus imaging

For immunohistochemistry, anesthetized Sprague–Dawley rats (P21) were transcardially perfused with 4% paraformaldehyde, and the excised brain was immersed in the fixative at 4 °C overnight before embedding in paraffin blocks and sectioned in a microtome. Sections were deparaffinized and rehydrated in xylene and ethanol following standard protocols prior to labeling (primary antibody: anti-NKCC1, 1:400, Abcam AB59791, secondary antibody: Alexa Fluor® Goat anti-rabbit IgG, 1:500, Life Tech A-11034). Sections were mounted with ProLong Gold DAPI mounting medium (Dako) and imaged using a Zeiss LSM700 point laser (Argon Lasos RMC781272) scanning confocal microscope with a Zeiss Plan-Apochromat 63 × /1.4 numerical aperture oil immersion objective (Carl Zeiss, Oberkochen, Germany).

## Results

**Fig. 4:**
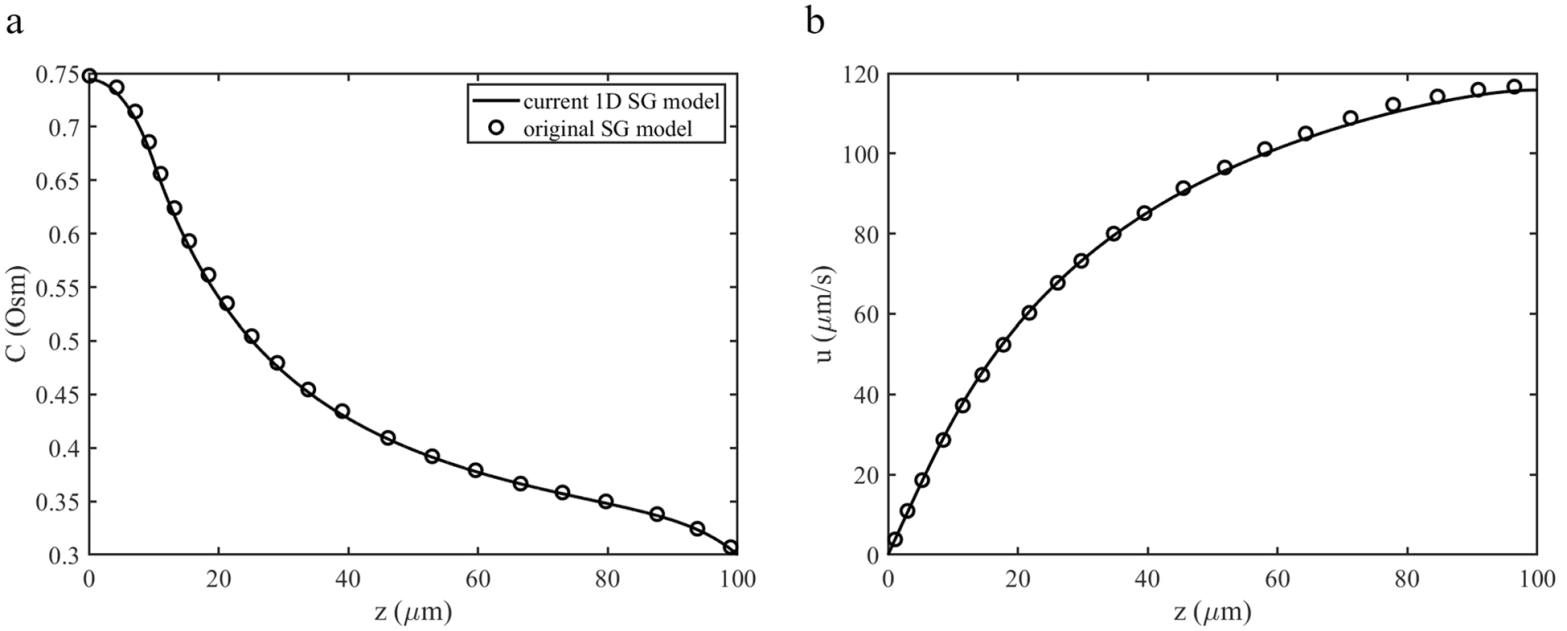
Comparison of model output between the current 1D SG model (solid line) with protected length set to zero and the original standing gradient model by Diamond and Bossert (open circles) using parameter values from [14] as listed in Table 1. Concentration (**a**) and the velocity (**b**) profiles along the FU z-axis are shown

To verify the implementation of the 1D SG model, we selected the same parameter values as used by Diamond and Bossert for their original standing gradient one [14]. Since the original model does not consider a protected region, we set the protected length to zero for this comparison. The solute flux was prescribed as a function of z-location in the FU to match the conditions in [14]. Similarly, the hydraulic diameter of the FU was directly set. Figure 4 shows that the velocity and concentration distributions along the FU z-axis of the two models match very well. This indicates that Eqs. (4) and (5) with the boundary conditions (6)–(8) were solved consistently.

**Fig. 5:**
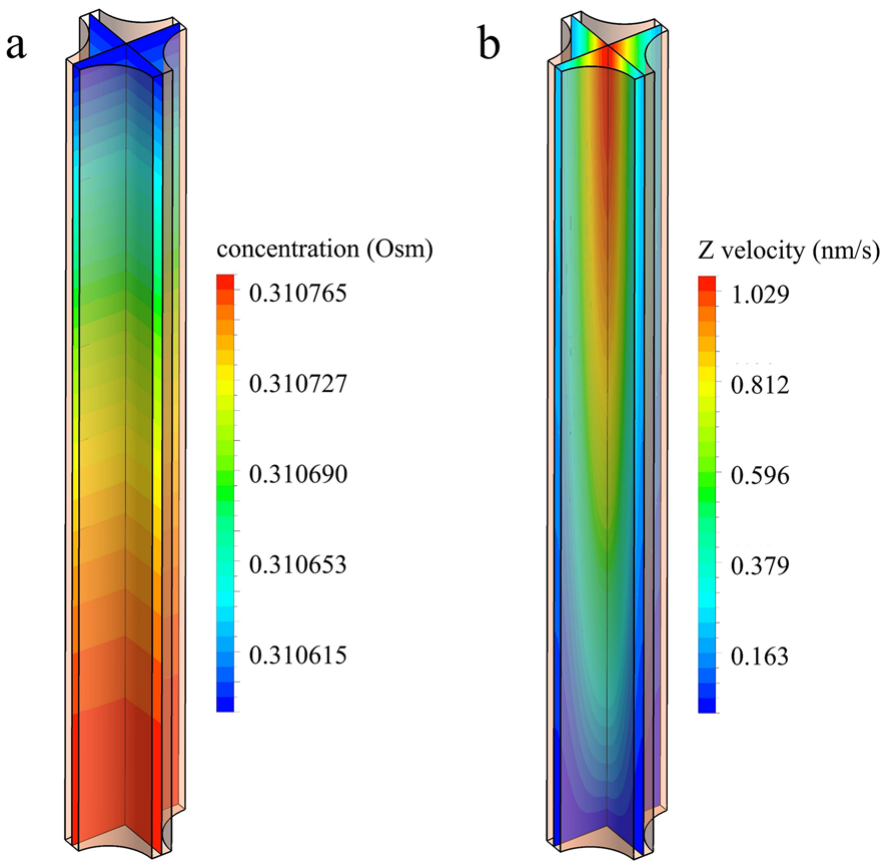
The profiles of concentration (**a**) and z-velocity (**b**) in a FU as computed with the 3D model. The contours are shown on two perpendicular planes passing through the center line of the FU

We assessed the applicability of the 1D SG model for testing the hypothesis that standing osmotic gradients are the main drivers of CSF production in rats by comparing its production rate predictions with those of the more intricate 3D SG model. The set of parameter values given in Table 3 was used to this end. Figure 5 shows the concentration and z-velocity distribution in the FU on two section planes as computed with the 3D model for the mean case. The concentration field appears largely one-dimensional (varying only along the z-axis), while the velocity is distributed three-dimensionally with the maximum at the center of the FU. Table 4 lists the production rates predicted by the 1D and 3D models, showing that the 1D model reports higher CSF production rates by the SG mechanism than the 3D model predicts.

**Table 4:** CSF production rates calculated by the 3D and 1D models with the parameter values given in Table 3

Case	3D model (µL/min)	1D model (µL/min)	1D/3D ratio (–)	Active surface ratio (–)
Mean − SD	0.0013	0.0025	1.886	0.502
Mean	0.0053	0.0067	1.273	0.781
Mean + SD	0.0073	0.0109	1.494	0.651

A larger 1D/3D ratio indicates a stronger overestimation of CSF production by the 1D model. The active surface ratio is defined as the surface area through which solutes enter the FU divided by the overall FU surface area

**Fig. 6:**
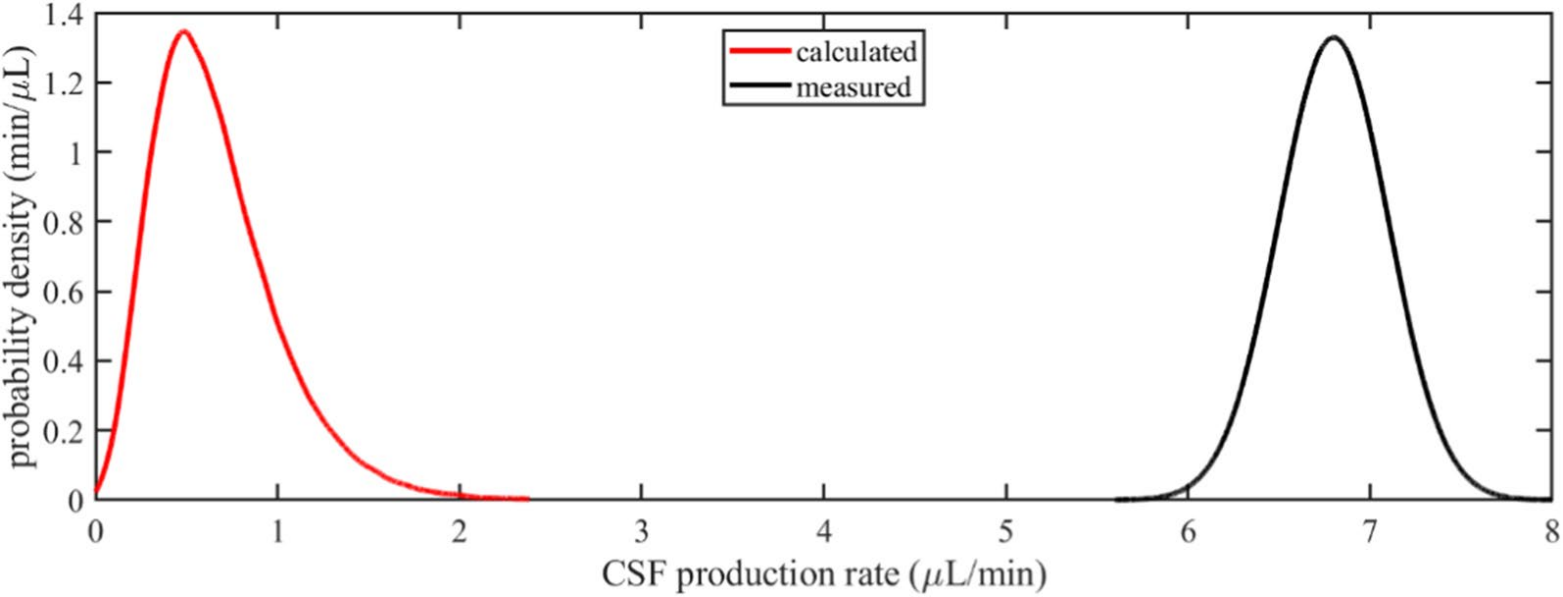
Probability density functions of measured CSF production values (black) and calculated (red) CSF production by the standing osmotic gradient mechanism

After having verified the implementation of the 1D SG model and having shown that it likely over- rather than underestimates CSF production by the SG mechanism, we employed it to calculate the CSF production rate based on the parameter distributions given in Table 2. Figure 6 shows the probability density functions of the experimentally measured CSF production rate, and of the production rate attributed to the SG mechanism as per the 1D model. The latter amounts to less than 10% of the measured total CSF production rate. We note that the experimental measurements reflect actual CSF production resulting from all contributing driving forces, while the model provides an estimate of production by the SG mechanism alone. We will argue in the next section that the calculated CSF production by SG can be considered an upper limit, and why its actual contribution to overall CSF production is likely lower.

## Discussion

We aimed to test the hypothesis that standing osmotic gradients at the surface of the ChP epithelium are the main drivers of CSF production. To this end, we extended and reformulated the original standing gradient model, which was designed for absorptive epithelia, to reflect the conditions on the ChP epithelial surface. Upon verification of this new 1D SG model with the results of Diamond and Bossert [14], we compared its output with that of a new 3D SG model, showing that the 1D model attributes a higher CSF production rate to the SG mechanism for the same conditions. In other words, the 1D model likely overestimates the effect of standing osmotic gradients, which is of advantage for the purpose of plausibility testing. Finally, by performing approximately 200,000 calculations using the 1D model with different parameter value sets randomly selected based on the likelihood of each value, we found that the predicted CSF production rate by the SG mechanism, if it were the sole contributor to CSF production, is one order of magnitude below that of the actual CSF production rate. This suggests that potential inter-microvillar standing osmotic gradients, even when enhanced by macroscopic stagnant pools, are not sufficient to drive CSF production by the ChP.

Both models require the prescription of solute flux into the FU. Since solute flux into the inter-microvillar space has not been experimentally quantified, we assumed that all solutes in the ventricular space originate in inter-microvillar spaces, and that all solutes exit the ventricles driven by bulk CSF flow. Under steady-state conditions, ventricular CSF production is equal to the amount of CSF leaving the ventricles. Therefore, the cumulative solute flux from all FUs corresponds to the bulk solute concentration multiplied by the CSF production rate. This means that the actual CSF production rate is an input to the models, and the calculated production rate corresponds to the fraction that can be attributed to the SG mechanism. Since there is a wide range of measured CSF production rates in rats reported in the literature [6, 30–33], we repeated the probabilistic 1D model calculations for another value of the measured CSF production rate that marks the lower end of this range, namely 0.74 ± 0.05 µL/min [31]. We note that the nominal value used marks the upper end with 6.8 ± 0.3 µL/min (Table 2). The production rate calculated with the lower end was 0.07 ± 0.05 µL/min, thus still one order of magnitude smaller than the measured rate. This behavior is expected, since a reduction in the prescribed CSF production rate lowers solute flux into the FUs, which reduces trans-epithelial osmotic gradients. The prescription of solute flux into the FU in this manner likely leads to an overestimation of CSF production via standing gradients by both models, since the inter-microvillar spaces do not have to be the sole source of solutes.

Neither the 1D nor the 3D model accounts for ChP motion, which could be caused by cardiovascular and respiratory action or by head and spine movement. Any such motion would work against CSF production by the SG mechanism both by disturbing standing gradients in the inter-microvillar spaces and by flushing pools of protected CSF from ChP folds. In the 1D model, the effect of protected pools is considered by the protected length, which is defined purely based on anatomic considerations. To obtain more accurate predictions of the CSF production rate, the effective protected length should be considered instead, noting that its value is location-dependent and difficult to determine. For the purposes of this study, it is sufficient to note that the values of protected length considered here are higher than the corresponding effective values. To understand the impact of changes in protected length on CSF production, we simplified Eqs. (6)–(8) using a small Péclet number approximation (see Additional file 1), which yielded a linear relation between the production rate and protected length: an increase in the protected length while maintaining other parameters constant yields a corresponding linear increase in CSF production. Thus, while not actively contributing to CSF production, protected pools may amplify the effect of potential standing gradients in the inter-microvillar space. Since the protected length values used here are expected to be larger than the effective ones, the model likely overestimates CSF production by the SG mechanism.

The 1D model predicts a higher CSF production rate than the 3D model. This can be attributed to a difference in the area ratio of the FU solute input surface (active surface) to its total surface. In the 1D model, solutes enter the FU through the whole side surface (Fig. 1d), whereas in the 3D model, parts of the side surfaces are interfaces to neighboring FUs, i.e., no solute flux occurs through those. Consequently, solutes that diffuse in the circumferential direction and produce standing gradients away from the active surfaces cannot contribute to CSF production. This effect is not accounted for in the 1D model, where the ratio of active to total surface is equal to one. Table 4 shows that the overestimation of CSF production by the 1D model reduces as the active to total surface ratio in the 3D model increases. Consequently, it is reasonable to assume that the 1D model generally predicts a higher CSF production rate by the SG mechanism than the 3D model.

The transport processes in the ventricular space, and in particular around the ChP, are complex. They include both diffusive and advective modes, moving interfaces, and transient CSF dynamics. Neither the 1D nor the 3D model can capture the effect of all processes potentially relevant to the SG mechanism. However, the models are designed to overestimate CSF production by standing osmotic gradients. This is achieved by making simplifying assumptions on some of the model parameters: we supposed that all solutes in the ventricular space originate in the ChP inter-microvillar spaces, which leads to an overestimation of the standing gradients. Neglecting tissue and bulk fluid motion (that would disturb standing gradients) results in a larger protected length. Assuming an active to total surface ratio of one for the FU results in a high transmembrane water transport rate. Finally, considering the SG mechanism as the sole driving force for CSF production discounts the effects of other mechanisms, such as hydrostatic pressure gradients, that would reduce standing gradients. Consequently, the here-reported contribution of the SG mechanism to ChP CSF production likely constitutes an upper limit.

## Conclusion

We have implemented and verified a one-dimensional standing osmotic gradient model of CSF production on the ChP luminal surface that also accounts for the effect of protected pools in ChP folds. Calculations with this model, based on probabilistic parameter value distributions derived from experimental measurements, suggest that potential local osmotic gradients in the inter-microvillar spaces are too small to contribute substantially to CSF production; this even though the underlying model assumptions, which include protected pools of CSF, favor the SG mechanism. ChP motion and movement of CSF in the ventricles, both not accounted for in the model, would reduce local osmotic gradients, making it unlikely that they are the main drivers of CSF production.

## Supplementary Information

**Additional file 1.** Derivation of the functional unit tip boundary condition, estimation of choroid plexus surface area and luminal membrane permeability, Péclet number calculation, and estimation of the protected length

## Data Availability

Code and data supporting the conclusions of this study are available on request from the corresponding author.

## Abbreviations

1D: One-dimensional
3D: Three-dimensional
CSF: Cerebrospinal fluid
ChP: Choroid plexus
FU: Functional unit
NBCe2: Na^+^-HCO_3_^−^ cotransporter
NKCC1: Na^+^-K^+^-2Cl^−^ cotransporter 1
SD: Standard deviation
SG: Standing gradient
