# Supplementary material for: Are standing osmotic gradients the main driver of cerebrospinal fluid production? A computational analysis
Source: Fluids Barriers CNS. 2023 Mar 13;20:18. doi: 10.1186/s12987-023-00419-2 (PMC10012606; doi:10.1186/s12987-023-00419-2)
Supplement: Supplementary file 1 — Additional file 1. Derivation of the functional unit tip boundary condition, estimation of choroid plexus surface area and luminal membrane permeability, Péclet number calculation, and estimation of the protected length [file 12987_2023_419_MOESM1_ESM.pdf]

Additional File 1

Derivation of the FU Tip Boundary Condition

The protected zone is an open area (without any membrane) where solute transport is governed by the advection-diffusion equation (S1). Note that there is no CSF production in the protected zone, but uniform velocity results from CSF entering from the FU. The general form of the solute concentration profile in the protected region is given by Equation (S2), where α_1, α_2, and u are unknown. The continuity of the concentration profile everywhere necessitates equal concentration value (C) and slope ($\mathrm{d}C/\mathrm{d}z$) at the interface of the FU and the protected region ($z = l_{\mathrm{mv}}$). At the ventricular end of the protected region, the concentration is equal to the bulk CSF concentration (Equation (S3)), and at its FU end, the value and the slope are equal to the corresponding ones inside the FU (Equations (S4) and (S5)). Subtracting Equation (S3) from (S4) and substituting α_2 into Equation (S5) yields Equation (8).

$$D\frac{\mathrm{d}^2C}{\mathrm{dz}^2} - u\frac{\mathrm{d}C}{\mathrm{dz}} = 0 \qquad \text{(S1)}$$

$$C(z) = \alpha_1 + \alpha_2 e^{\frac{u}{D}z} \qquad \text{(S2)}$$

$$\alpha_1 + \alpha_2 e^{\frac{u}{D}(l_{\mathrm{mv}} + l_{\mathrm{prot}})} = C_0 \qquad \text{at } z = l_{\mathrm{mv}} + l_{\mathrm{prot}} \qquad \text{(S3)}$$

$$\alpha_1 + \alpha_2 e^{\frac{u}{D}l_{\mathrm{mv}}} = C\big|_{z=l_{\mathrm{mv}}} \qquad \text{at } z = l_{\mathrm{mv}} \qquad \text{(S4)}$$

$$\alpha_2 \frac{u}{D} e^{\frac{u}{D}l_{\mathrm{mv}}} = \frac{\mathrm{d}C}{\mathrm{d}z}\bigg|_{z=l_{\mathrm{mv}}} \qquad \text{at } z = l_{\mathrm{mv}} \qquad \text{(S5)}$$

Estimation of Choroid Plexus Surface Area and Luminal Membrane Permeability

The apparent area of the ChP, A_{app}, cannot be measured easily because of its convoluted surface, but it can be estimated from its weight as

$$A_{\text{app}} = \frac{e.m}{\rho_e.h} \tag{S6}$$

where, e, m, h, and ρ_{e} are the ChP epithelial ratio, ChP mass, cell height, and the tissue density. The value distribution of these parameters is shown in Table S1. The luminal membrane permeability, L_{p}, can also be derived from other parameters as

$$L_{\text{p}} = \frac{2}{A_{\text{app}}(1 + 2\pi r_{\text{mv}} l_{\text{mv}} \sigma)} \left(\frac{Q_{\text{meas}}}{\Delta C}\right) \tag{S7}$$

Here, ΔC is the transepithelial concentration difference (between blood and CSF in the ventricular space). Note that the epithelial resistance to water transport is assumed to be equally divided between luminal and basolateral membranes.

Parameters	Symbol	mean	SD	Reference
Epithelial ratio (-)	e	0.878	0.025	[27]
ChP mass (mg)	m	4.69	0.33	[26]
Cell height (μm)	h	11.3	0.5	[27]
Tissue density (g/cm^3)	ρ_{e}	1	0.04	[28]
Measured CSF production rate change with transepithelial concentration difference (μL/min·mOsm)	$\frac{Q_{\text{meas}}}{\Delta C}$	0.022	0.008	[6]

Table S1. Mean and standard deviation (SD) of parameters used in Equations (S6) and (S7) with the corresponding references. Normal distribution was assumed.

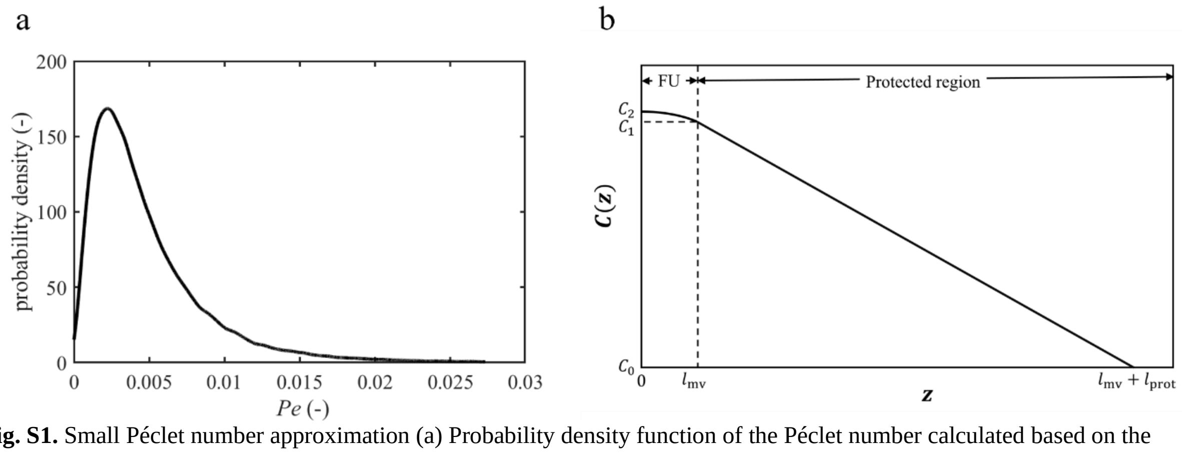

Fig. S1. Small Péclet number approximation (a) Probability density function of the Péclet number calculated based on the parameter value distributions reported in Table 2. (b) A schematic distribution of concentration along a typical FU and protected zone with parametric representation of the main points.

Péclet Number Calculation

The Péclet number (Pe) is a measure for the relevance of convection compared to diffusion for solute transport. Figure S1a shows that the Péclet number, defined as $Pe = u|_{z=l_{\mathrm{mv}}} \cdot l_{\mathrm{prot}}/D$, is very small for all possible parameter permutations. As a result, solute transport in the FU and the protected region is dominated by diffusion, and the small Pe approximation for Equations (6) – (8) can be applied. In this case, Equation (8) reduces to Equation (S8). Considering the solute flux balance on the boundaries of the FU and applying the conditions in Equations (6) and (7) yields Equation (S9). Combining Equations (S8) and (S9) results in Equation (S10), which shows a linear relation between concentration drop along the protected region and l_{prot}.

$$\left.\frac{\mathrm{d}C}{\mathrm{d}z}\right|_{z=l_{\mathrm{mv}}} = \frac{C_0 - C_1}{l_{\mathrm{prot}}} \tag{S8}$$

$$u_1 C_1 - D\left.\frac{\mathrm{d}C}{\mathrm{d}z}\right|_{z=l_{\mathrm{mv}}} = \frac{4}{\rho d}\int_0^{l_{\mathrm{mv}}} \phi(z)\mathrm{d}z \tag{S9}$$

$$C_1 - C_0 = \frac{4}{\rho d D} l_{\mathrm{prot}} \int_0^{l_{\mathrm{mv}}} \phi(z)\mathrm{d}z \tag{S10}$$

Here, u_1 and C_1 are, respectively, the velocity and concentration on the interface between FU and the protected region (Fig. S1b). The production rate Q_{p} is calculated from

$$Q_{\mathrm{p}} = N\int_0^{l_{\mathrm{mv}}} L_{\mathrm{p}}(C(z) - C_0)(\pi d)\mathrm{d}z, \tag{S11}$$

which can be decomposed into two terms:

$$Q_{\mathrm{p}} = N\int_0^{l_{\mathrm{mv}}} L_{\mathrm{p}}(C(z) - C_1)(\pi d)\mathrm{d}z + N\int_0^{l_{\mathrm{mv}}} L_{\mathrm{p}}(C_1 - C_0)(\pi d)\mathrm{d}z \tag{S12}$$

Given that l_{prot} is generally two orders of magnitude larger than l_{mv}, it follows that

$$C_1 - C_0 \gg C_2 - C_1 > C(z) - C_1, \tag{S13}$$

i.e., the second term in Equation (S12) has a much stronger contribution to the production rate (see Fig. S1b) and the first one can be neglected. Here, C_2 denotes the concentration at the base of the FU. Thus, the production rate is approximately linearly related to the protected length as well:

$$Q_{\text{p}} \cong \frac{4\pi N L_{\text{p}} l_{\text{mv}} \int_0^{l_{\text{mv}}} \phi(z)\text{d}z}{\rho D} l_{\text{prot}} \tag{S14}$$

To assess the validity of the approximated model, Equation (S14) was used to calculate the CSF production rate for the same parameter permutations used in the full 1D model. Figure S2a shows that for parameter permutations that result in small CSF production rates, predictions of the full and approximated 1D model match well. At higher production rates, the approximated model overpredicts CSF production (Fig. S2b).

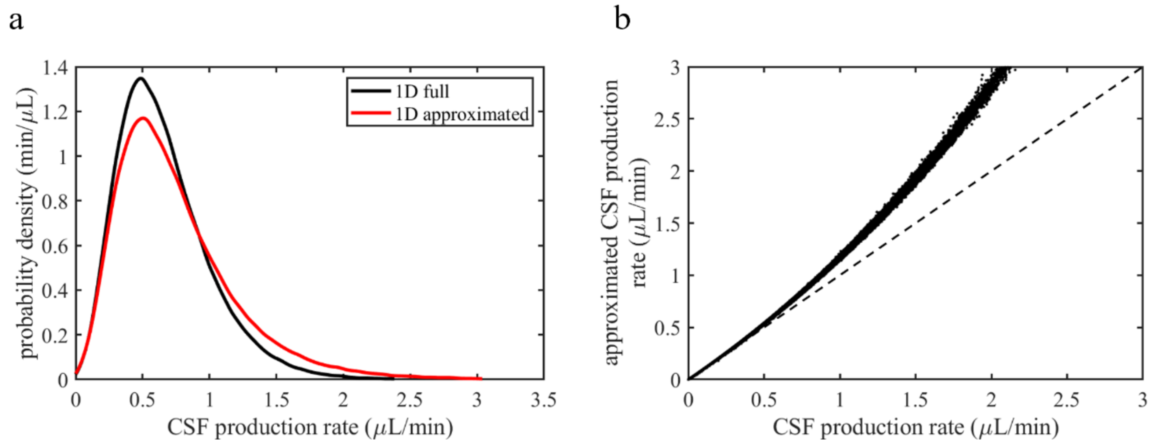

Fig. S2. Comparison of the full 1D model results (in black) with those of its small *Pe* approximation (in red). (a) Probability density of the predicted CSF production rate using the same parameter permutations. (b) CSF production rates predicted by the full 1D model plotted against those of its approximation. Perfect correlation between the two would follow the black dashed line.

Estimation of the Protected Length

As shown in the magnified view in Fig. 3c, the folds of ChP consist of two regions: the intermicrovillar space and the protected region. CSF pools of the protected region are separated from the bulk CSF flow by a virtual surface enveloping the ChP. The exact distribution of the protected length could be determined precisely from the shape of the luminal ChP surface and the envelope surface (see Fig. 3d), but this would require currently unavailable *in vivo* high-resolution scans of the entire rat ChP. As a workaround, the average protected length can be estimated from available data.

The average protected length is calculated as

$$\langle l_{\mathrm{prot}} \rangle = \frac{\sum_{j=1}^{n_{\mathrm{f}}} \sum_{i=1}^{m^{(j)}} l_{\mathrm{prot}_i}^{(j)}}{N}, \tag{S15}$$

where N is the total number of FUs on the ChP surface. We assume that the ChP has n_{f} folds, each one having $m^{(j)}$ FUs. Each FU is located at a distance $l_{\mathrm{prot}_i}^{(j)}$ (corresponding to the ith FU in the jth fold) away from bulk CSF. We note that $\sum_{j=1}^{n_{\mathrm{f}}} m^{(j)}$ is smaller than N since some FUs are located outside of folds (corresponding to zero protected length). These FUs do not contribute to the nominator of Equation (S15). We define λ as the fraction of the ChP surface in contact with the envelope surface (where the protected length is zero). Then, $1-\lambda$ is the fraction of the ChP surface within folds, i.e., where the protected length is greater than zero. For a graphical interpretation of λ, Fig. S3 shows three artificial ChP morphologies for which the fraction of ChP in contact with the envelope varies from 0 to 1.

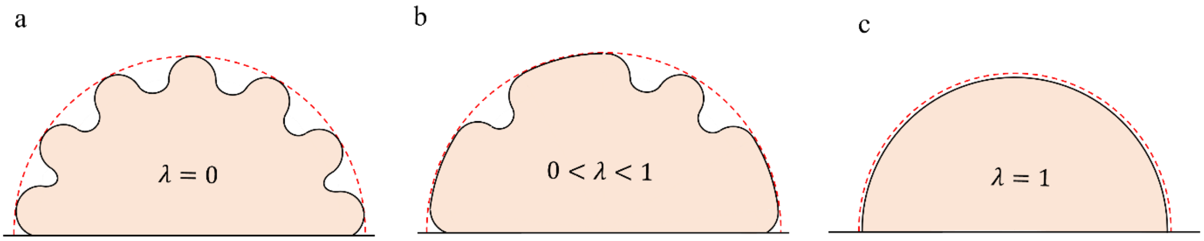

Fig. S3. Illustration of the geometric relation between the ChP surface and its envelope expressed by λ. The black solid and red dashed curves show the ChP and envelope surfaces, respectively. (a) Brush-like ChP, where the envelope contacts the ChP surface at a limited number of infinitesimally small points. (b) Generic ChP shape in which the envelope and luminal surfaces coincide at some locations. (c) Semi-circular ChP shape with no protected region (the ChP surface coincides with the envelope surface).

We use δA_{ChP} to designate the area of a ChP surface patch covering one FU, and δA_{env} the area of the juxtaposed envelope patch. Therefore, for each fold, the number of envelope and

surface patches is equal (see Fig. 3 in the paper). The value of δA_{ChP} is the same for all FUs (since uniform FUs were used in the models) and thus for all folds. δA_{env} is chosen to be constant within each fold. Multiplying both the nominator and the denominator of Equation (S15) with δA_{ChP}, we obtain

$$\langle l_{\mathrm{prot}} \rangle = \frac{\sum_{j=1}^{n_{\mathrm{f}}} \sum_{i=1}^{m^{(j)}} l_{\mathrm{prot}_i}^{(j)}}{N \cdot \delta A_{\mathrm{ChP}}} = \frac{\sum_{j=1}^{n_{\mathrm{f}}} \sum_{i=1}^{m^{(j)}} l_{\mathrm{prot}_i}^{(j)} \cdot \left(\frac{\delta A_{\mathrm{ChP}}}{\delta A_{\mathrm{env}_i}^{(j)}} \right) \cdot \delta A_{\mathrm{env}_i}^{(j)}}{A_{\mathrm{ChP}}} \tag{S16}$$

The term $(\delta A_{\mathrm{ChP}} / \delta A_{\mathrm{env}_i}^{(j)})$ is 1 where the ChP surface and its envelope coincide, and >1 in the folds, since there the ChP has to cover a larger area than the envelope. This ratio may be different for each fold, since the envelop patch area depends on the fold shape. We now geometrically rearrange the individual folds to merge them into one large fold without changing the protected lengths of the individual FUs. This is permissible since maintaining the protected lengths ensures that the CSF production does not change. Also, it does not change the average protected length according to Equation (S16). The envelope area of the combined single fold is made up of patches of different sizes. We now scale the individual envelope patch areas to one average area, $\delta\overline{A_{\mathrm{env}}}$, and reformulate Equation (S16) accordingly:

$$\langle l_{\mathrm{prot}} \rangle = \frac{\sum_{j=1}^{n_{\mathrm{f}}} \sum_{i=1}^{m^{(j)}} l_{\mathrm{prot}_i}^{(j)} \cdot \left(\frac{\delta A_{\mathrm{ChP}}}{\delta\overline{A_{\mathrm{env}}}} \right) \cdot \delta\overline{A_{\mathrm{env}}}}{A_{\mathrm{ChP}}} \tag{S17}$$

Using the globally constant ChP surface patch area and the average envelope patch area, we can now relate the patch area ratio to global ChP parameters:

$$\frac{\delta A_{\mathrm{ChP}}}{\delta\overline{A_{\mathrm{env}}}} \cong \frac{(1-\lambda) A_{\mathrm{ChP}}}{A_{\mathrm{env}} - \lambda A_{\mathrm{ChP}}} \tag{S18}$$

The numerator on the right-hand side corresponds to the ChP surface area in the fold, while the denominator is the area of the envelope covering the fold. Inserting Equation (S18) in Equation (S17), we obtain a new expression for the average protected length:

$$\langle l_{\mathrm{prot}} \rangle = \frac{\frac{(1-\lambda)A_{\mathrm{ChP}}}{A_{\mathrm{env}}-\lambda A_{\mathrm{ChP}}}\left[\sum_{j=1}^{n_{\mathrm{f}}}\sum_{i=1}^{m^{(j)}} l_{\mathrm{prot}_i}^{(j)} \cdot \delta\overline{A_{\mathrm{env}}}\right]}{A_{\mathrm{ChP}}} \qquad \text{(S19)}$$

Since $\delta\overline{A_{\mathrm{env}}}$ is smaller than or equal to δA_{ChP}, the term $l_{\mathrm{prot}_i}^{(j)} \cdot \delta\overline{A_{\mathrm{env}}}$ is always smaller than the unit volume of the protected region (the blue rectangles shown in Fig. 3c). Hence, the expression in brackets in Equation (S19) is always smaller than the volume of the protected region. By replacing the expression with the total volume of protected regions, which is equal to the volume enclosed by the envelope, V_{env}, minus the total volume of the ChP, V_{ChP}, we obtain an upper limit for the average protected length:

$$\langle l_{\mathrm{prot}} \rangle \leq \frac{(1-\lambda)(V_{\mathrm{env}}-V_{\mathrm{ChP}})}{A_{\mathrm{env}}-\lambda A_{\mathrm{ChP}}} \qquad \text{(S20)}$$

The shape of the ChP is partially accounted for by λ. For example, if the ChP does not fold (Fig. S3c), λ is equal to one and the right-hand side of Equation (S20) becomes zero, indicating that the average protected length is zero.

To compute Equation (S20) for the actual ChP geometry, the envelope surface area is required. A_{env} cannot be larger than the inner surface area of the ventricles, A_{vent}. In the hypothetical situation where a) $\lambda = 0$, and b) the ChP fills the entire ventricular space, i.e., V_{env} is equal to the ventricular volume, V_{vent}, and $A_{\mathrm{env}} = A_{\mathrm{vent}}$, the protected CSF pools reach their largest possible size. Therefore,

$$\langle l_{\mathrm{prot}} \rangle \leq \frac{V_{\mathrm{vent}}-V_{\mathrm{ChP}}}{A_{\mathrm{vent}}} \qquad \text{(S21)}$$

Using the Sprague Dawley rat brain atlas (v4, RRID: SCR_017124) [25], we measured the total volume and area of the ventricular space to be 34.1 mm^3 and 380 mm^2, respectively. The ChP volume is 4.69 mm^3 (Table S1). Thus, based on Equation (S21), the average protected length is ≤ 77.5 µm. We selected this upper limit of 77.5 µm as the center value of a normal distribution of the average protected length. Therefore, the l_{prot} model parameter space extends to 155 µm, which is favorable for CSF production by the SG mechanism.
